# The Mighty Fruit Fly Moves into Outbred Genetics

**DOI:** 10.1371/journal.pgen.1006388

**Published:** 2016-11-03

**Authors:** Ewan Birney

**Affiliations:** European Molecular Biology Laboratory, European Bioinformatics Institute (EBI), Wellcome Trust Genome Campus, Hinxton, Cambridge, United Kingdom; Stanford University School of Medicine, UNITED STATES

January 2016’s issue of *PLOS Genetics* has an outbred genetics study in body size traits [1], perhaps one of the most studied parts of human genetics, but in a somewhat surprising organism—fruit flies (*Drosophila melanogaster*). I will return below to both the motivation for using laboratory organisms for outbred genetics and the opportunities this presents, but let’s first briefly walk through this interesting study.

Vonesch and colleagues studied 143 inbred lines (DRGP) that had been drawn from a wild *D*. *melanogaster* population inbred by Trudy Mackay and colleagues in North Carolina. Each line has been inbred for at least 20 generations, making the majority of their genome homozygous, and (with the caveat of removal of lethal recessive alleles) the homozygous haplotype is a draw from the original population. They measured 26 different body size measurements—the fly equivalent of height, hip-to-waist ratio, and leg length—though of course they sound far more exotic given that this is fly anatomy, such as wing shape partitions and interocular distance (a measure of head size). The DRGP has been fully genome sequenced, so the authors performed a relatively straightforward association study of alleles in the population to these quantitative phenotypes. One locus was significant even with stringent statistical correction for multiple testing. So far, this study is very similar to human studies except for the very low sample number compared to the massive anthropometric studies in humans.

But what Vonesch and colleagues do next, one cannot do in humans; they make use of the extensive RNAi libraries in Drosophila, combined with the precise tissue-specific activation, and could show that ~65% of the weaker hits, below traditional significance levels, were validated by this orthogonal genetic perturbation (a random set RNAi knock down shows around 40% of genes changing wing size). Interestingly, only the first locus (*kek1*) is a “traditional” growth gene discovered by the forward genetics approach of screening mutagenized flies for easily distinguishable phenotypes; the other loci can be linked to growth pathways by interaction maps, but many of them are in uncharacterized genes in the fly. Furthermore, the measurement of the same autosomal genotype in the two different sexes allowed them to characterize sex-specific effects.

This study therefore reveals a different component of body size control in Drosophila compared to forward genetics—and also links some unknown genes in Drosophila to pathways, thus chipping away at another aspect of metazoan biology. But this is also an excellent example of using laboratory animals to understand outbred genetics. The Arabidopsis community has been making extensive use of both wild and managed populations to help understand the basic biology of flowering plants; the DRGP along with other resources in Drosophila population genetics are now coming of age, with this paper being one of a number of phenotyping papers coming out (for example, Ayroles and colleagues [2]). There are many benefits of these studies. By having a renewable resource of the same genotypic individuals, but those individuals being drawn from a wild population, one does experiments that require large numbers of individuals (for example, molecular studies needing cell numbers) or studies the variance between individuals in an isogenic background. Furthermore, as this renewable resource can be shared between investigators, one can directly correlate phenotypic measurements (of all sorts) between laboratories. As *D*. *melanogaster* is arguably the best studied metazoan in the world, there is a massive body of knowledge on the function of many genes, derived from a range of developmental, phenotypic, and molecular routes. Drosophila have an extensive set of molecular tools available, a short generation time, and a collaborative community of lines and resources; a well-tooled up Drosophila lab can design and execute all manner of experiments in months compared to years for mice and of course many experiments that are unfeasible in humans.

Drosophila is not the only organism with these types of accessible outbred panels. Plant biologists have been making such wild derived and other cultivar panels across many species (Arabidopsis [3], Maize [4]); the large scale collaborative cross [5] in mice provides a pseudo-outbred population in the prime experimental mammal; I am part of a collaboration to establish a wild derived panel of Medaka fish [6] with similar properties. But Drosophila is furthest along in terms of metazoans, largely due to the vision of Trudy Mackay, and is being exploited by a broad group of researchers looking at many different phenotypes.

In many ways, the revival of human genetics in the last decade has seen the realization of the early pioneering genetic work in the 1920s, back then motivated by both human and organismal genetics. We now have the ability to genotype and phenotype at scale on all sorts of creatures, ourselves included. However, there can be a myopic view that our ability to scale up in humans makes most other animal outbred genetic studies redundant—why not simply measure everything we want in the organism we principally want to understand—namely humans? But this greatly overestimates our understanding of how variation in genotype leads to phenotypic changes, in particular for interactions between genes and with the environment. We have far better control of genotype and environment in other organisms, allowing us to develop well powered studies to look at epistasis or environment interactions, using phenotypes such as developmentally restricted times that are impossible in the human context, and unlike humans we can immediately take the discoveries back into the very same organism to validate. The ability to critically test models with specific experiments is particularly appealing. The statistical models to unpick epistasis or environment interactions are unsurprisingly becoming more and more complex, but we need to have systems where we can control all the variables to validate that the entire approach works before we trust them fully in the less controlled and less experimentally accessible human populations.

This is just the start of these studies—there are many more studies on DRGP happening in the community. We do need to coordinate the resulting data better than the current hodgepodge of supplementary tables, and this will allow both better reanalysis and integrative studies between laboratories. For example, in this paper the authors focused on only 2 measurements rather than using all 26; more powerful multiphenotype approaches may well extract more power from this study. Furthermore, the current size of DRGP is limiting—in all populations, most variants are at low frequency, and low frequency variants are likely to be far more interesting in terms of their fitness effects. Thankfully, there are moves to expand the DRGP to around 1,000 lines. The fruit fly was the cornerstone of early genetics and provided many breakthroughs in developmental biology via forward genetics. Outbred genetics in well-controlled conditions can now be added to this humble organism’s contribution to the field.
